# *Panax notoginseng* Promotes Repair of Colonic Microvascular Injury in Sprague-Dawley Rats with Experimental Colitis

**DOI:** 10.1155/2018/4386571

**Published:** 2018-01-29

**Authors:** Shiying Wang, Ping Tao, Lei Zhao, Wangjun Zhang, Hongyi Hu, Jiang Lin

**Affiliations:** ^1^Department of Gastroenterology, Longhua Hospital, Shanghai University of TCM, Shanghai, China; ^2^Institute of Digestive Diseases, China-Canada Center of Research for Digestive Diseases (ccCRDD), Longhua Hospital, Shanghai University of TCM, Shanghai, China

## Abstract

To investigate the therapeutic effects of PN on intestinal inflammation and microvascular injury and its mechanisms, dextran sodium sulfate- (DSS-) or iodoacetamide- (IA-) induced rat colitis models were used. After colitis model was established, PN was orally administered for 7 days at daily dosage of 1.0 g/kg. Obvious colonic inflammation and mucosal injuries and microvessels were observed in DSS- and IA-induced colitis groups. DAI scores, serum concentrations of VEGFA121, VEGFA165, VEGFA165/VEGFA121, IL-6, and TNF-*α*, and expression of Rap1GAP and TSP1 proteins in the colon were significantly higher while serum concentrations of IL-4 and IL-10 and MVD in colon were significantly lower in the colitis model groups than in the normal control group. PN promoted repair of colonic mucosal injury and microvessels, attenuated inflammation, and decreased DAI scores in rats with colitis. PN also decreased the serum concentrations of VEGFA121, VEGFA165, VEGFA165/VEGFA121, IL-6, and TNF-*α* and increased the serum concentrations of IL-4 and IL-10, with the expression of Rap1GAP and TSP1 proteins in colonic mucosa being downregulated. The constituents of PN were identified with HPLC-DAD. To sum up, PN could promote repair of injuries of colonic mucosa and microvessels via downregulating VEGFA isoforms and inhibiting Rap1GAP/TSP1 signaling pathway.

## 1. Introduction

Colonic mucosal ulcers and increased intestinal permeability are the major pathological features of UC [1]. Colonic microvascular injury has been widely observed in UC patients and considered as an important pathogenesis of UC in recent years [2]. Mucosal microvessels injury accompanied with increased vascular permeability [3, 4] results in the imbalance between oxygen consumption and oxygen delivery in colonic mucosa [5]. In addition, our previous study found that colonic mucosal damage was significantly improved when mucosal microvessels injury was repaired in rats with experimental colitis [6]. Therefore, we speculate that microvascular injury may play an important role in the pathogenesis of UC and may be a new target in UC treatment.

*Panax notoginseng* (PN), a traditional Chinese medicine used to treat vascular lesions, was found to promote repair of colonic mucosal damage in UC [7, 8]. PN has various bioactive components. Some of them, such as Notoginsenoside R1 and Ginsenoside Rg1, promote growth and proliferation of vascular endothelial cell (VEC) and play an important role in maintaining the structure and the function of blood vessels [9, 10]. Although PN has been widely used to treat UC in recent years and has an obvious effect on repairing colonic mucosa damage [8], its mechanism is still unclear Therefore, we hypothesized that PN could promote repair of mucosal damage via regulating angiogenesis.

## 2. Materials and Methods

### 2.1. Animals

Sprague-Dawley (SD) rats (120~140 g) were purchased from Shanghai SLAC Laboratory Animal, Co., Ltd. (Shanghai, China). The Institutional Animal Care and Use Committee of Shanghai University of Traditional Chinese Medicine approved all procedures (number SZY201612006). Rats were housed in a pathogen-free environment and allowed to acclimate to the environment for 7 d before inclusion in an experiment.

### 2.2. Preparation of PN

PN was purchased from Shanghai Huayu Traditional Chinese Medicine Co., Ltd. (Shanghai, China). PN (0.9 kg) was dispersed in tap water (6 L), boiled at 100°C for 3 h, filtered through a sieve (150 *μ*m), extracted with absolute ethanol, and dried in a freeze dryer. The brown-colored extract powder of PN was stored at −20°C.

### 2.3. Identification of Phytochemical Constituents

A Shimadzu LC system (Shimadzu, Kyoto, Japan) was employed for liquid chromatographic analysis. The LC system was connected by a hybrid ion trap/time-of-flight mass spectrometer equipped with an electrospray ionization (ESI) source to perform high-resolution tandem mass spectrometry. HPLC analysis was conducted on a Jade-Pak ODS-AQ column (250 × 4.6 mm, 5 *μ*m, TechWay). The mobile phase (1.0 mL/min) consists of aqueous acetonitrile (A)—0.1%—with formic acid (B) with a gradient program as follows: 0–27 min, 18.5%–23% A; 27–50 min, 23%–21% A; 50–53 min, 21%–29% A; 53–110 min, 29%–37% A. Roughly 25% portion of the effluent was introduced into the ESI source by splitting the effluent via two PEEK tubes with length ratio of 1 : 3. A detection wavelength of 203 nm and an injection volume of 10 *μ*L were set. Optimization of the operating conditions and the equipment and the reagents for HRMS analysis were identical to those used in the previous study [11].

### 2.4. IA- and DSS-Induced Colitis

Two rat models of colitis were used in this study. One was induced by 0.1 mL 6% IA (Sigma-Aldrich, MO, USA) dissolved in 1% methylcellulose given to rats once by enema (7 cm above the anus) via rubber catheter Nelaton S-8 (Rüsch, Germany). The other was induced by DSS (Sigma-Aldrich, MO, USA): rats were allowed free access to purified water containing 5% DSS (w/v) for 7 d. DSS solution was prepared daily as previously reported [12].

### 2.5. PN Intervention In Vivo

SD rats were divided into normal control group (*n* = 6), experimental colitis model control group (*n* = 6), and PN intervention group (*n* = 6). For the intervention groups, PN was intragastrically administered to rats with IA-induced or DSS-induced colitis once daily for seven consecutive days at the dosage of 1.0 g/kg. For the control groups, normal saline was given to the corresponding rats. The initial administration times of PN were 3 d after establishing the colitis models. After 7 days of treatment, the rats were sacrificed for pathological evaluation, vascular permeability analysis, and cytokines detection. Three independent experiments were performed in triplicate.

### 2.6. Disease Activity Index (DAI)

The severity of colitis was evaluated with DAI, as described previously [13]. The parameters of DAI included weight loss (0, none; 1, 0–5%; 2, 5–10%; 3, 10–20%; 4, >20%); stool consistency (0, none; 2, loose stool; 4, watery); and bleeding (0, none; 1, trace; 2, mild occult blood; 3, obvious occult blood; 4, gross bleeding). DAI was scored before, during, and after the treatment.

### 2.7. Histological Assay

Segments of colon were fixed in formalin buffer and embedded in paraffin. Sections 5 *μ*m thick were deparaffinized and stained with hematoxylin and eosin (H&E). The histological changes were assessed by a pathologist.

### 2.8. Microvessel Density (MVD) Analysis

5 *μ*m thick paraffin-embedded sections of colon were deparaffinized, subjected to heat-mediated antigen retrieval, and blocked with goat serum. The tissues were incubated with the primary anti-CD31 antibody (1 : 200, v/v) (Abcam, UK) overnight at 4°C. After three 5 min washes, the horseradish peroxidase- (HRP-) labeled secondary antibody (1 : 300) was added and the samples were incubated at 37°C for 1 h. The sections were counterstained with hematoxylin for 1 min at room temperature to visualize the endothelial cell nuclei. Three fields with CD31-positive cells in each section were chosen to assess MVD. The MVD value was calculated as the average vessel counts in three selected areas within a microscopic field.

### 2.9. Vascular Permeability (VP) in Rats

VP of vessels in colonic mucosa was evaluated by the Evans blue method, as described previously [14]. Rats were anesthetized with intraperitoneal injection of sodium pentobarbital. Evans blue (1 mg/100 g) (Sigma-Aldrich, MO, USA) was injected intravenously 15 min before autopsy. Evans blue was extracted from the 1 cm segment of colonic tissue using formamide and measured by spectrophotometry at 610 nm. Results were expressed as OD value per milligram of colon.

### 2.10. Enzyme-Linked Immunosorbent Assay (ELISA)

Blood samples were collected from the abdominal aorta. The serum concentrations of VEGFA165, VEGFA121 (Cloud-Clone Corp, TX, USA), IL-4 (R&D, MN, USA), IL-6 (R&D, MN, USA), IL-10 (R&D, MN, USA), and TNF-*α* (R&D, MN, USA) were detected using the appropriate ELISA kits. Absorbance was measured on a microtitre plate reader.

### 2.11. Western Blotting

Rap1GAP and TSP1 proteins were measured by western blotting as described previously [6]. Colonic tissue was cut into pieces and homogenized in fivefold volumes of ice-cold homogenizing buffer (0.1 mmol/L NaCl, 0.1 mol/L Tris-HCl, and 0.001 mol/L EDTA) containing 1 mmol/L phenylmethylsulfonyl fluoride, 1 mg/mL aprotinin, and 0.1 mmol/L leupeptin at 3000 ×g and 4°C for 1 h. Bovine serum albumin was used to estimate the protein content in supernatants. The protein samples (60 *μ*g in each sample) were subjected to SDS-PAGE and transferred to polyvinylidene fluoride membranes using a transfer apparatus (Bio-Rad, Hercules, CA, USA). The membranes were blocked for 2 h; then the primary antibodies anti-Rap1GAP (1 : 1000) and anti-TSP1 (1 : 1000) were added and incubated at 4°C overnight, and the corresponding HRP-conjugated secondary antibody (Cell Signaling Technology) was added and incubated for 1 h. Protein-antibody complexes were detected by Clarity Western ECL Substrate (Bio-Rad), and results were authenticated with the Image J software (Gene Co., Ltd., China).

### 2.12. Statistical Analysis

Data were presented as the mean ± SD. One-way ANOVA or general linear model with repeated measures was used to analyze the data sets with three or more groups and least significant difference post hoc test for multiple comparisons. Student's *t*-test was used to analyze data sets with two groups. *P* < 0.05 was considered significant.

## 3. Results

### 3.1. The Main Phytochemical Constituents in the PN

**Figure 1:**
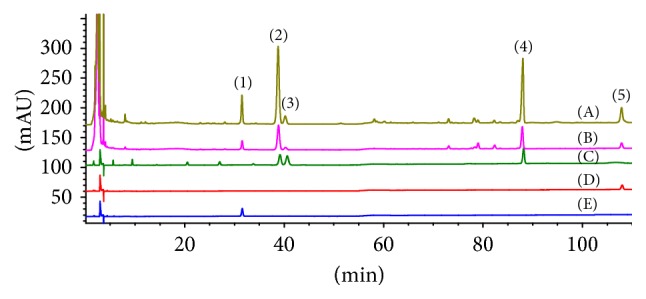
*The HPLC analysis of the extract of Panax notoginseng*. (A) Exact of PN; (B)* notoginseng* formula particles (allowed for using in clinic, Lot: A1500376); (C) standards sample 1^*∗*^ (Ginsenoside Rg1, Ginsenoside Re, and Ginsenoside Rb1); (D) standards sample 2 (Ginsenoside Rd); (E) standards sample 3 (Notoginsenoside R1). Notoginsenoside R1 (1) was detected at approximately 33.001 minutes, Ginsenoside Rg1 (2) was detected at approximately 39.175 minutes, Ginsenoside Re (3) was detected at approximately 40.632 minutes, Ginsenoside Rb1 (4) was detected at approximately 88.182 minutes, and Ginsenoside Rd (5) was detected at approximately 107.994 minutes. ^*∗*^They are hard to be completely purified.

Five saponins were found in PN via comparing the retention times of the isolated compounds in HPLC chromatogram and analyzing MZ data. These five saponins were Notoginsenoside R1 (0.094 mg/mL, retention time (RT): 31.571 min), Ginsenoside Rg1 (0.15264 mg/mL, RT: 39.175 min), Ginsenoside Re (0.17744 mg/mL, RT: 40.632 min), Ginsenoside Rb1 (0.24384 mg/mL, RT: 88.182 min), and Ginsenoside Rd (0.11857 mg/mL, RT: 107.994 min), respectively (Figure 1). The structures of these five saponins were described in previous study [4].

### 3.2. The Effects of PN on Colonic Mucosal Inflammation

**Figure 2:**
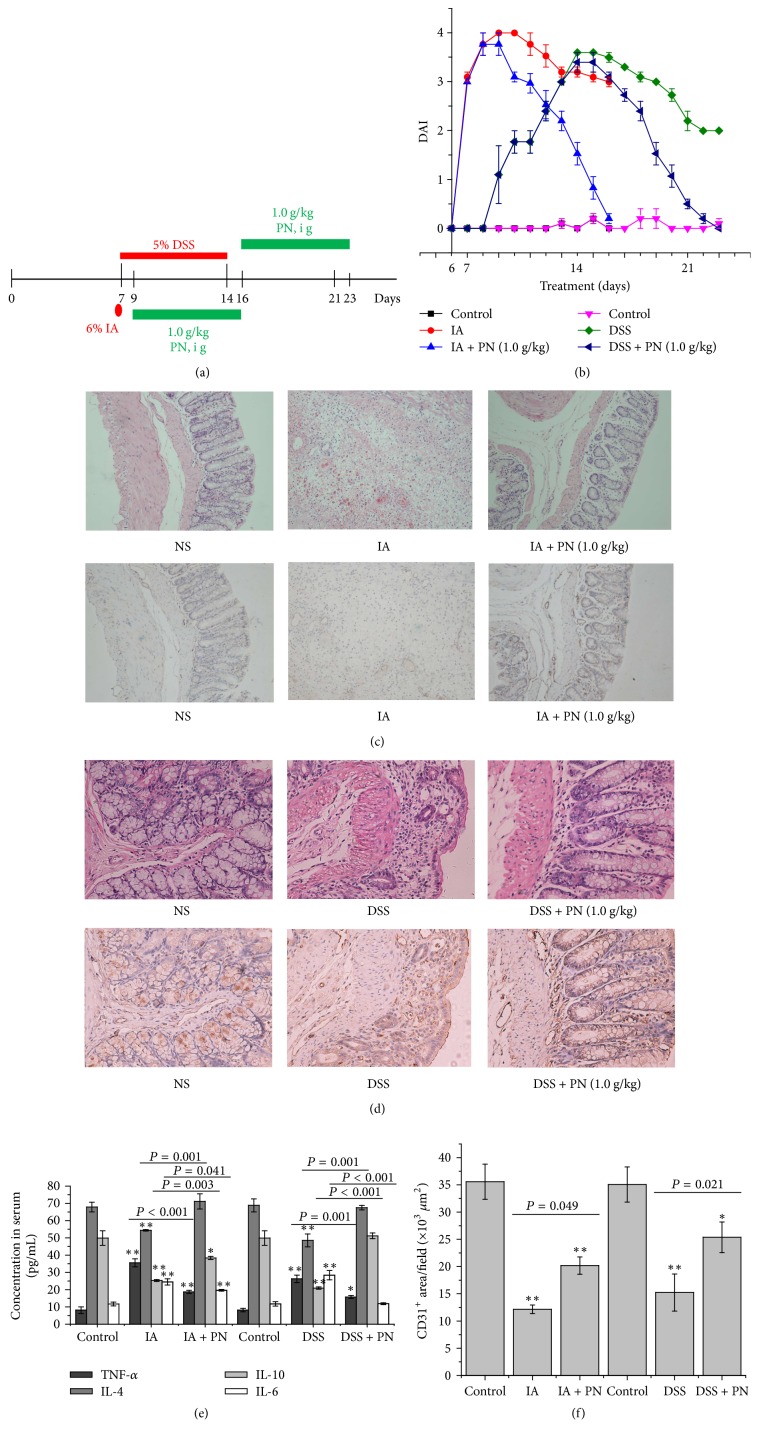
*The efficacy of PN on colonic microvascular injury and mucosal damage*. (a) Experimental induction of UC and treatment. (b) Profile of DAI, (c, d, f) histopathological examination of the colon (magnification, ×400) and MVD, (e) serum levels of TNF-*α*, IL-4, IL-6, and IL-10 in the study. All data, expressed as means ± standard error of the mean (SEM) of three independent experiments performed in triplicate, were analyzed by one-way ANOVA and Student's *t*-test (^*∗*^*P* < 0.05, ^*∗∗*^*P* < 0.01).

Compared with the normal control group, DAI scores were much higher in the DSS- and IA-induced experimental colitis control groups (Figure 2(b)). Obvious hyperemia and inflammatory cells infiltration were found in the colitis control groups (Figures 2(c) and 2(d)) accompanied with higher proinflammatory cytokines (IL-6 and TNF-*α*) and lower anti-inflammatory cytokines (IL-4 and IL-10) (Figure 2(e)). After being treated by PN, DAI scores were decreased, hyperemia and inflammatory cells infiltration were attenuated, proinflammatory cytokines were downregulated, and anti-inflammatory cytokines were upregulated in the rats with colitis. These results suggested that PN could attenuate the inflammation and improve repair of mucosal injury induced by DSS or IA.

### 3.3. The Effects of PN on Mucosal Microvascular Injury and Vascular Permeability

**Figure 3:**
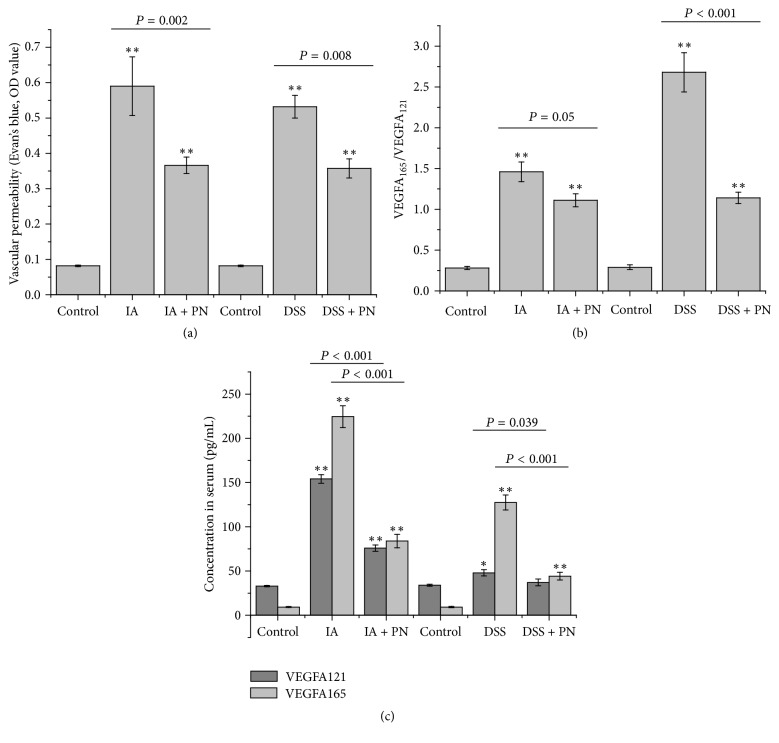
*PN reversed the vascular permeability (VP) in colon and the disordered serum ratio of VEGFA165/VEGFA121*. (a) Profile of VP, (b, c) serum levels of VEGFA165 and VEGFA121, and the ratio of VEGFA165/VEGFA121 in the study. All data, expressed as means ± SEM of three independent experiments performed in triplicate, were analyzed by one-way ANOVA and Student's *t*-test (^*∗*^*P* < 0.05, ^*∗∗*^*P* < 0.01).

Compared with the normal control group, the MVD values were significantly decreased and vascular permeability was significantly increased in the colitis control groups (Figures 2(f) and 3(a)). However, after treatment by PN, the MVD values were increased while the vascular permeability was decreased in the rats with colitis. These results indicated that PN might improve repair of mucosal microvascular injury.

### 3.4. PN Reversed the Disordered Serum Ratio of VEGFA165/VEGFA121

The serum concentrations of VEGFA165 and VEGFA121 and the serum ratio of VEGFA165/VEGFA121 were significantly increased in IA- and DSS-induced experimental colitis groups compared with the normal control group. After treatment by PN, the increased serum concentrations of VEGFA165 and VEGFA121 were downregulated and the disordered serum ratio of VEGFA165/VEGFA121 was reversed in the rats with colitis (Figures 3(b) and 3(c)).

### 3.5. PN Inhibited Rap1GAP/TSP1 Signaling Pathway

**Figure 4:**
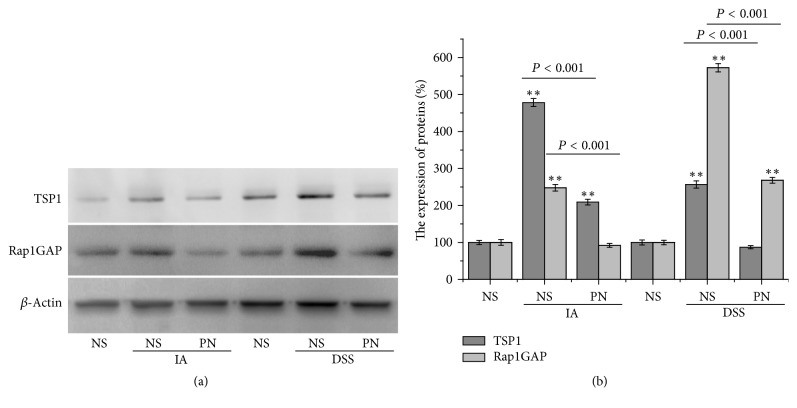
*PN blocked Rap1GAP/TSP1 signaling pathway*. (a, b) The expression level of Rap1GAP and TSP1 proteins in colonic mucosa in rats. All data, expressed as means ± SEM of three independent experiments performed in triplicate, were analyzed by one-way ANOVA and Student's *t*-test (^*∗∗*^*P* < 0.01).

The expression of Rap1GAP and TSP1 proteins was significantly increased in IA- and DSS-induced experimental colitis groups compared with the normal control group. After treatment by PN, the expression of Rap1GAP and TSP1 proteins in colonic mucosa was reversed in the rats with colitis (Figures 4(a) and 4(b)).

## 4. Discussion

PN is a Chinese herbal medicine with multiple pharmacological effects and widely used to treat cardiovascular diseases, pain, inflammation, and hemorrhagic injuries [15]. The bioactive components and their contents in PN are different depending on the place of origin and the season of collection. In order to evaluate the quality of PN, we used high performance liquid chromatography (HPLC) to analyze the composition of PN used in this study. The main components of PN in this study included Notoginsenoside R1, Ginsenoside Rg1, Ginsenoside Re, Ginsenoside Rb1, and Ginsenoside Rd. The components and their contents are in line with the provisions of the Chinese Pharmacopoeia.

It was found that significant microvascular injury occurred in the early stage of UC onset and it might be important for promoting the development of UC [3]. The oxygen supply in colonic mucosa is dependent on the number and the function of microvessels in mucosa. When inflammation occurs in colonic mucosa, it causes increased oxygen consumption and malfunction of microvessels which results in decreased oxygen supply [5, 16]. The imbalance of oxygen supply and oxygen consumption causes hypoxia and apoptosis of endothelium cells which aggravates the mucosa injury [5, 17]. It was also found in the previous study that PN could promote repair of mucosal ulcer and reduce inflammatory response in patients with inflammatory bowel diseases (IBD) including ulcerative colitis (UC) and Crohn's disease (CD) [18]. This suggested that PN might repair mucosal ulcer via attenuating microvascular injury. Proangiogenic therapy has been considered as a new challenge for gastrointestinal ulcers [19].

However, the effect of PN on regulating the angiogenesis is still controversial. Qiao et al. found that PN extract could inhibit angiogenesis of atherosclerotic plaque by downregulating VEGF [20]. On the contrary, Yang et al. found PN had the effects of proangiogenesis and antiapoptosis [21]. Therefore, to further clarify the effect of PN on angiogenesis is critical to its clinical application. Our results showed that the degree of mucosal inflammation and damage was closely related to the degree of vascular injuries. After treatment by PN, the microvascular injury and the mucosal damage were attenuated in the experimental colitis, which was similar to the previous study [8].

Although many putative cytokines have been considered as regulators of angiogenesis, VEGF, especially VEGFA, plays most important role in angiogenesis [22, 23]. Both VEGFA121 and VEGFA165, two isoforms of VEGF, are closely related to the development of colonic microvessels. We also found that the increased ratio of VEGFA165/VEGFA121 was correlated with the decreased numbers of microvessels [6]. In this study, increased VEGFA121 and VEGFA165 were downregulated by PN in rats with colitis accompanied with attenuation of microvascular injury.

In addition, our results showed in previous study that Rap1GAP/TSP signaling pathway was important in regulating the development of colonic microvessels [6]. The increasing expression of Rap1GAP and TSP1 proteins had a close relationship with the damage of mucosa and microvessels in colon. With the expression of Rap1GAP and TSP1 proteins inhibited, microvascular injury was decreased in colon. In the study, the expression of Rap1GAP and TSP1 proteins was significantly downregulated by PN in colonic mucosa with the microvascular injury decreased in the rats with colitis.

## 5. Conclusions

In summary, PN could downregulate increased VEGFA121 and VEGFA165 and block Rap1GAP/TSP1 signaling pathway in experimental colitis so that it attenuates microvascular injury, which resulted in improving repair of mucosal injury. There are still some issues unsolved in this study. How does PN regulate VEGFA121 and VEGFA165? Which component of PN plays the critical role in angiogenesis regulation? These are important for using PN to treat mucosal injury and need further exploring.

## Abbreviations

PN: * Panax notoginseng*
UC: Ulcerative colitis
HPLC-DAD: High performance liquid chromatography-diode array detection
DSS: Dextran sodium sulfate
IA: Iodoacetamide
SD: Sprague-Dawley
MVD: Microvessel density
VEGF: Vascular endothelial growth factor
IL: Interleukin.
